# TNFAIP6 defines the MSC subpopulation with enhanced immune suppression activities

**DOI:** 10.1186/s13287-022-03176-5

**Published:** 2022-09-24

**Authors:** Lingyun Li, Lei Yang, Xian Chen, Xiangjuan Chen, Lianghui Diao, Yong Zeng, Jianyong Xu

**Affiliations:** 1 Department of Immunology, School of Medicine, Health Science Center, Shenzhen University, Nanhai Avenue 3688, Shenzhen, 518060 Guangdong People’s Republic of China; 2 Shenzhen Key Laboratory for Reproductive Immunology of Peri-Implantation, Shenzhen Zhongshan Institute for Reproduction and Genetics, Fertility Center, Shenzhen Zhongshan Urology Hospital, Shenzhen, 518060 Guangdong People’s Republic of China; 3 Department of Obstetrics, Shenzhen University General Hospital, Shenzhen University, Shenzhen, People’s Republic of China

**Keywords:** Mesenchymal stromal/stem cells, MSCs, TNFAIP6, Tumor necrosis factor alpha-induced protein 6, Immune suppression

## Abstract

**Background:**

Mesenchymal stromal/stem cells (MSCs) have been intensively investigated in both pre-clinical and clinical studies. However, the therapeutic efficacy varies resulting from the heterogenicity of MSCs. Therefore, purifying the specific MSC subpopulation with specialized function is necessary for their therapeutic applications.

**Methods:**

The large-scale RNA sequencing analysis was performed to identify potential cell markers for the mouse MSCs. Then, the immune suppression activities of the purified MSC subpopulation were assessed in vitro and in vivo.

**Results:**

The TNFAIP6 (tumor necrosis factor alpha-induced protein 6) has been identified as a potential cell marker for mouse MSCs, irrespective of tissue origin and laboratory origin. The TNFAIP6^+^ mouse MSCs showed enhanced immune suppression activities and improved therapeutic effects on the mouse model of acute inflammation, resulting from faster response to immune stimulation.

**Conclusions:**

Therefore, we have demonstrated that the TNFAIP6^+^ MSC subpopulation has enhanced immune suppression capabilities.

**Supplementary Information:**

The online version contains supplementary material available at 10.1186/s13287-022-03176-5.

## Introduction

Mesenchymal stromal/stem cells (MSCs) have been intensively investigated in both pre-clinical and clinical studies, because of their multi-potent differentiation and immune modulation capabilities [1–4]. It has been demonstrated that the MSCs or MSC-derived exosomes have therapeutic effects on many diseases [2, 3, 5–7]. Unfortunately, the consistency of their therapeutic efficacy is very low, resulting from the heterogenicity of MSCs [1–3, 8]. Plenty of factors would induce the MSC heterogenicity, such as tissue origin, isolation methods, expansion medium, and the donor’s genetic background, gender, age, and healthy conditions [1, 4, 8–10].

Therefore, delineating the underlying mechanisms and developing novel strategies to target the heterogenicity are necessary to develop MSC-based therapies. Previously, we have developed several strategies to reduce the heterogenicity of MSCs, such as genetic modification [6, 11] and full chemical medium development [5, 12]. On the other hand, purifying specific MSC subpopulations for specific therapeutic purposes is also critical for improving the therapeutic efficacy and consistency, although this is very challenging [1].

Plenty of mouse MSC markers have been demonstrated, including cell surface markers (such as CD105^+^CD90^−^, CD90^+^, CD73^+^, LepR^+^, PDGFRα^+^, PDGFRα^+^Sca-1^+^, CD140b/PDGFRβ^+^, CD51^+^, Lgr6^+^, Meflin^+^, EphA7^+^, CD49f^high^, SSEA-4^+^, p75NTR^+^, CD44^+^, CD49e^+^, CD29^+^, and Sca-1^+^)[13–33], intracellular markers (such as Gli1, Nestin, Mx1, αSMA, Axin2, Ctsk^+^Scx^+^, Hox11, and Hic1) [34–44], and extracellular/secreted markers (such as Gremlin1, and CXCL12) [45–47]. However, none of these markers are MSC specific. Furthermore, the major therapeutic contributors of the MSC are secreted proteins [2]. Therefore, purifying the specific MSC subpopulation with specialized function is necessary for their therapeutic applications.

In the current study, we performed large-scale RNA sequencing analysis and screened potential cell markers for the mouse MSCs. Our data here demonstrated that TNFAIP6 (tumor necrosis factor alpha-induced protein 6) is a potential cell marker for mouse MSCs, irrespective of tissue origin and laboratory origin. TNFAIP6 is a secreted glycoprotein (around 35kD) with strong immune inhibitory functions and has shown therapeutic effects on immune disorder related diseases, such as acute lung injury, acute pancreatitis, liver injury, colitis, peritonitis, subarachnoid hemorrhage, rheumatoid arthritis, diabetes, and myocardial infarction [48–62]. It is expressed mainly by immune cells and stromal cells, including MSCs, especially when stimulated by pro-inflammatory cytokines, such as TNF-α, IL-1β, IFN-γ, and LPS [57, 63–65]. And the expression level of TNFAIP6 has been developed as a predictor of the therapeutic effects of MSCs in vivo [66]. TNFAIP6 could inhibit neutrophil migration and T helper cell differentiation, promote M2 macrophage polarization and regulatory T cell differentiation, modify ECM, and enhance tight junction [51, 52, 54, 55, 58, 60, 64, 67–69]. And our data here showed that the TNFAIP6^+^ mouse MSCs have enhanced immune suppression activities and improved therapeutic effects in the mouse model of acute inflammation.

## Materials and methods

### RNA-seq dataset and analysis

The RNA-seq data used in the current study consist of 185 non-MSC samples from different tissues (Additional file 1: Table S1) and 15 MSC samples (Additional file 2: Table S2). The RNA-seq data were defined as MSC samples when they were described as MSCs in the database and also the corresponding studies (Additional file 2: Table S2), according to the minimal criteria for the MSCs [70]. While the cells were characterized as mature cells, pluripotent cells, or other type of adult stem cells (Additional file 1: Table S1), they were defined as non-MSC samples. The raw RNA-seq data were downloaded from NCBI website (National Center for Biotechnology Information), and analyzed by RSEM (v1.3.3) [71], bowtie 2 (v 2.4.2), and then GC content normalized with EDASeq (v2.28.0) [72], as described previously [12, 73]. The mouse reference genome (version mm39) was used for mapping and RSEM alignment. PCA (principal component analysis) was performed with princomp and ggplot2 packages in R (v 4.0.0). The RNA-seq data were combined together with Python (v3.8.5150.0), and the tSNE (t-distributed stochastic neighbor embedding) analysis was performed with the Seurat package in R (v 4.0.0). Differential gene expression was conducted with DESeq2 (v 1.34.0) in Bioconductor (v3.14). The GO (Gene Ontology), GO enrichment network, and KEGG (Kyoto Encyclopedia of Genes and Genomes) enrichment were analyzed with the clusterProfiler package (v3.0.4) in R (v 4.0.0).

### Mouse experiments

The C57BL/6 J mice (female, 8 weeks old) were purchased from the Guangdong Medical Laboratory Animal Center. The mice were maintained in specific pathogen-free conditions, and this study was approved by the Animal Research Ethics Committee of the School of Medicine, Shenzhen University (A20201241).

### Cell isolation from bone marrow, placenta, and fat

Cell isolation was performed as described before with modifications [5, 6, 74]. Briefly, the bone marrow (BM) cells were flushed out of femurs and tibias. Placenta and fat cells were isolated with 4 mg/ml collagenase B and 4 mg/ml dispase (Roche Life Science). Then, the isolated cells were subjected to single-cell RNA sequencing or maintained with DMEM/low glucose (Thermo Fisher Scientific) plus 10% FBS (Thermo Fisher Scientific), 10 ng/ml basic fibroblast growth factor (bFGF, PeproTech), 50 μg/ml ascorbic acid (Selleckchem), and antibiotics (Thermo Fisher Scientific) in cell culture dishes.

### Single-cell RNA-seq and analysis

Fresh isolated cells from bone marrow, placenta, and fat were filtered with 40 μm strainer (Corning) and resuspended by 0.04% BSA in HBSS (Thermo Fisher Scientific) at the concentration of 1 × 10^6^ cells/mL. Single-cell GEM (Gel Beads-in-Emulsion) was produced with 10 × Genomics Chromium platform according to the instructions. Finally, the libraries were sequenced with Illumina NovaSeq 6000 System (paired-end mode). Raw data processing, mapping, filtering, and UMI count matrix generation were conducted with 10xGenomics pipeline Cell Ranger (v2.1.0). Then, the gene-cell barcode matrix was further analyzed with Seurat package in R (v 4.0.0).

### Flow cytometry

Cell preparation, antibody staining, and flow cytometry were performed as described previously [5, 6]. Briefly, for cell surface protein staining, the freshly isolated cells were incubated with 5% FBS in PBS (Thermo Fisher Scientific) for 30 min, and then, the cells were stained with anti-TNFAIP6 (Cat. No. PA5-75,332, Thermo Fisher Scientific) for 60 min on ice, followed by 3 times washes, and then FITC-conjugated anti-rabbit IgG secondary (Cat. No. F-2765, Thermo Fisher Scientific) antibody for 30 min. For CD45 staining, the cells were stained with PE-conjugated rat anti-mouse CD45 (Cat. No. 567111, BD Biosciences) or PE Rat IgG2b, κ Isotype Control (Cat. No. 555848, BD Biosciences) for 30 min. For CD44 staining, the cells were stained with FITC-conjugated rat anti-mouse CD44 (Cat. No. 561859, BD Biosciences) or FITC Rat IgG2b, κ Isotype Control (Cat. No. 553988, BD Biosciences) for 30 min. For whole cell protein staining, the cells were fixed with 4% PFA (Paraformaldehyde, Sigma), permeablized with 0.2% Triton-X 100 in PBS with 10% FBS for 30 min, and then subjected to antibody staining. Cells were analyzed with BD AccuriC6 Plus (BD Biosciences), and the data were analyzed with FlowJo software.

### TNFAIP6^+^ cell purification

The freshly isolated BM cells were firstly subjected to CD45^+^ cell depletion with CD45 MicroBeads (mouse, Miltenyi Biotec) according to the instructions. Then, the CD45^−^ BM cells were stained the primary antibody to TNFAIP6 (Cat. No. PA5-75,332, Thermo Fisher Scientific). After staining the FITC-conjugated anti-rabbit IgG secondary (Cat. No. F-2765, Thermo Fisher Scientific) antibody for 1 h, the cells were sorted by BD FACSAria SORP cell sorter (BD Biosciences).

### Cell proliferation and death analysis

The cell doubling time was assessed as described before [12]. Briefly, the purified TNFAIP6^+^ or TNFAIP6^−^ mouse MSCs were plated onto p6 plates at the concentration of 10 × 10^4^ cells per well and expanded with the culture medium [6]. Cells were detached with TrypLE when they reached 80–90% confluence and counted with hemocytometer. The cell death was determined with the Cytotoxicity Detection Kit (Cat. No. 4744934001, Sigma) as described previously [5].

### ELISA

The TNFAIP6^+^ or TNFAIP6^−^ mouse MSCs were plated onto p12 plates at the concentration of 20 × 10^4^ cells per well. Three days later, the cell culture supernatant was collected and the protein level of TNFAIP6 was measured with mouse TNFAIP6 ELISA kit (Cat. No. CSB-EL023959MO, CUSABIO TECHNOLOGY) according to the instructions.

Peripheral blood was collected from the eyes of the mice, and the serum level of IL-6 (Cat. No. 431304), TNF-α (Cat. No. 430901), IFN-γ (Cat. No. 430801), and IL-1β (Cat. No. 432601) was measured with the ELISA kits (all from BioLegend) according to the instructions.

### Splenocytes and MSCs co-culture

The splenocytes were isolated and co-cultured with MSCs as described before with modifications [6]. Briefly, the splenocytes (20 × 10^4^ cells/well) isolated from 8-week-old C57BL/6 J mice were co-cultured with freshly purified TNFAIP6^+^ or TNFAIP6^−^ mouse MSCs (5 × 10^4^ cells/well) in 96-well plates with stimulation medium, which contained RPMI1640 plus 10% FBS and 1 × Cell Stimulation Cocktail (all from Thermo Fisher Scientific) for 48 h. Then, the cell proliferation was determined using the Cell Proliferation Kit I (Cat. No.11465007001, Roche), and measured by the automated microplate reader (model 550; Bio-Rad) at 570 nm. The CFSE measurement was performed with CellTrace™ CFSE Cell Proliferation Kit (Thermo Fisher Scientific) according to the instructions.

### MSC differentiation

The adipocytes, osteocytes, and chondrocytes differentiation and characterization were performed as previously described [6]. To quantify the adipocyte differentiation efficiency, the Oil Red O was eluted with isopropanol (200μL/well in p12 plate) for 30 min with agitation after lipid droplets staining, and measured by the automated microplate reader (model 550; Bio-Rad) at 490 nm. To quantify the osteocyte differentiation efficiency, the Alizarin Red S was eluted with 10% cetylpyridinium chloride (Sigma, prepared in PBS pH 7.5) at 37 ºC for 2 h with agitation after calcium staining, and measured by the automated microplate reader (model 550; Bio-Rad) at 405 nm. To quantify the chondrocyte differentiation efficiency, the Alcian Blue was eluted with 6 M guanidine hydrochloride for 3 h with agitation after glycosaminoglycan staining, and measured by the automated microplate reader (model 550; Bio-Rad) at 630 nm.

### RNA extraction and qPCR

The RNA extraction, cDNA synthesis, and real-time PCR were performed as previously described [6, 74]. The primer sequences used are shown in Additional file 3: Table S3.

### Mouse model of acute inflammation and cell transplantation

The endotoxin-induced acute inflammation mouse model was established as described previously [75]. Briefly, the C57BL/6 J mice (8 weeks old; 20 ± 1 g) were intraperitoneally injected a single dose of 20 mg/kg LPS (Lipopolysaccharides, Cat. No. L5293, Sigma). The TNFAIP6^+^ or TNFAIP6^−^ mouse MSCs (1 × 10^6^ cells/mouse) were transplanted intraperitoneally 30 min before the LPS injection. Each group contained 8 mice.

### Lung analysis

Bronchoalveolar lavage (BAL) was performed with cannulating the trachea, washing 3 times with 1 mL PBS containing 0.4 mM EDTA and protease inhibitor mixture (Cat. No. 87786, Thermo Fisher Scientific). After centrifugation at 700 g for 5 min, the supernatant was used for cytokine analysis. The total cell, neutrophil (CD45^+^CD11b^+^Ly-6G^+^Ly-6C^med^), and CD45^+^ cells in BAL were measured by flow cytometry. The myeloperoxidase (MPO) activity was quantified with the MPO Activity Assay Kit (Fluorometric, Cat. No. ab111749, Abcam) according to the instructions.

### Histological analysis

The lung tissue collection, fixation, tissue processing, embedding, sectioning, and HE (hematoxylin and eosin) staining were performed as described previously [6]. For CD45 staining, the sections were permeablized with 0.2% Triton-X 100 in PBS with 10% FBS for 30 min, followed by rabbit anti-mouse CD45 (Cat. No. ab10558, Abcam) incubation overnight, and detected with the SABC (Rabbit IgG)-POD kit (Cat. No. SA0021, Solarbio).

### Statistics

Data were analyzed with SPSS software for Windows (SPSS Inc.) and are shown as mean ± SEM (standard error of the mean). Two groups comparison was analyzed with Student’s *t* test. Multiple groups comparison was analyzed with 1-way ANOVA with normal data distribution, parametric test, and Tukey post hoc tests. *P* < 0.05 was considered statistically significant.

## Results

**Fig. 1:**
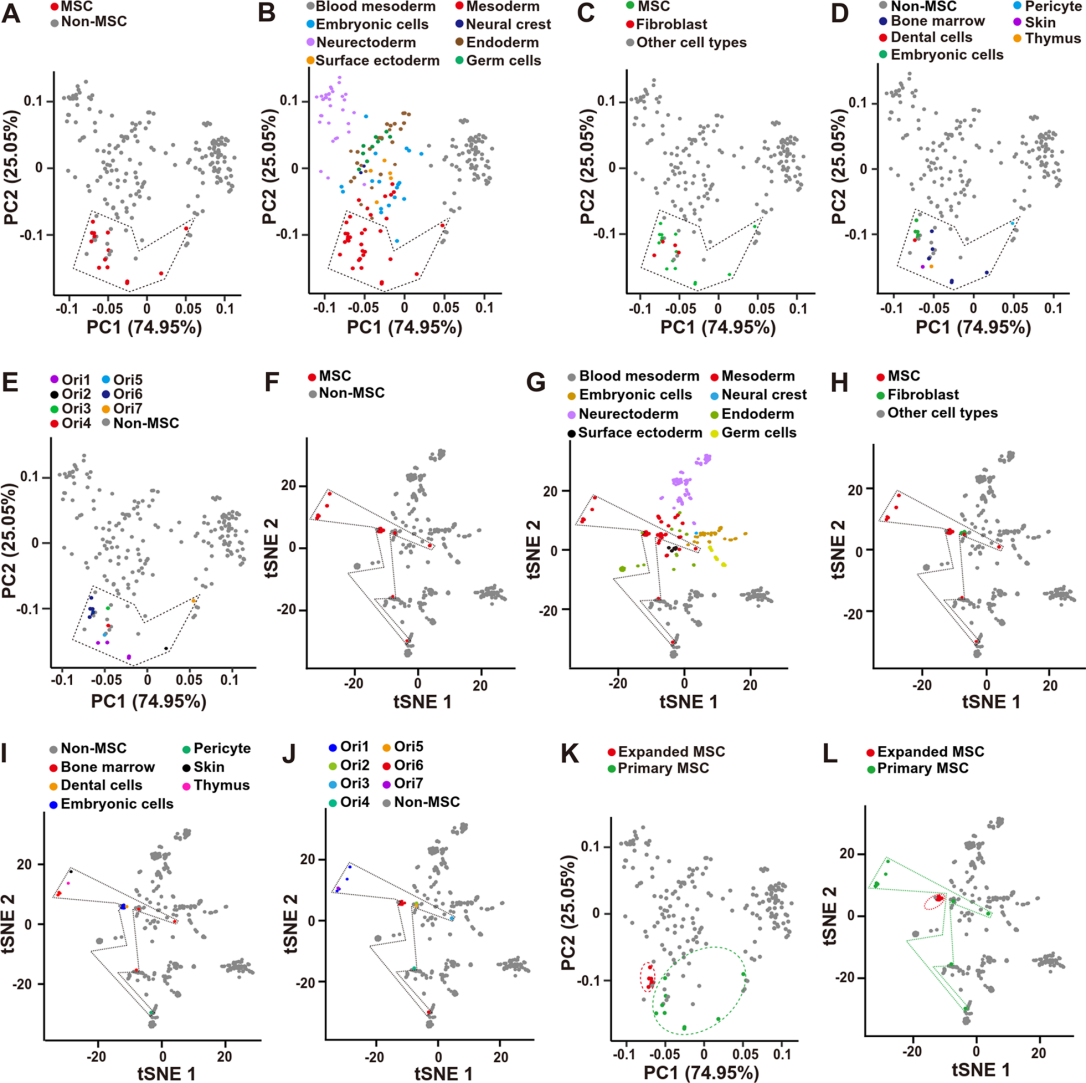
Bioinformatic analysis of MSCs and non-MSCs RNA-seq data. **A** PCA analysis of MSCs and non-MSCs transcriptome. Red dot indicates MSC samples and they were framed with dash lines. **B** Different germ lineages were labeled in PCA analysis. **C** Fibroblasts (red dot) and MSCs (green dot) were labeled in PCA analysis. **D** MSCs from different tissue origins were labeled in PCA analysis. **E** MSCs from different laboratory origins were labeled in PCA analysis. **F** MSCs and non-MSCs transcriptome was analyzed with tSNE. Red dot indicates MSC samples and they were framed with dash lines. **G** Different germ lineages were labeled in tSNE analysis. **H** Fibroblasts and MSCs were labeled in tSNE analysis. **I** MSCs from different tissue origins were labeled in tSNE analysis. **J** MSCs from different laboratory origins were labeled in tSNE analysis. **K** Expanded and primary MSCs were labeled in PCA analysis. **L** Expanded and primary MSCs were labeled in tSNE analysis. *MSCs* mesenchymal stromal/stem cells; *Ori* laboratory origin

To identify the potential cell marker of the mouse MSCs, which is universal expressed irrespective of tissue origin, development stage, and processing procedures, the total RNA sequencing data were downloaded from NCBI database (https://www.ncbi.nlm.nih.gov/), consisting of different mouse tissues and different types of MSC from different laboratories and tissues (Additional file 1: Table S1 and Additional file 2: Table S2). PCA analysis with R packages showed that most of the MSC samples clustered together (Fig. 1A) and they belonged to the mesoderm lineage (Fig. 1B). However, when comparing the MSCs with fibroblasts, they did not separate into two different populations (Fig. 1C), indicating that they might share some transcriptome similarities or the MSCs were not carefully characterized. Then, the tissue and laboratory origin was labeled (Fig. 1D, E, Additional file 2: Table S2). Although the MSCs from different tissues had different transcriptome (Fig. 1D), the laboratory origin contributed more to the heterogenicity of MSCs, indicating that the isolation and expansion procedures induced more heterogenicities (Fig. 1E). Then, the RNA sequencing data were integrated together and re-analyzed with another algorithm, tSNE (t-distributed stochastic neighbor embedding). The tSNE analysis showed the similar pattern to the PCA analysis (Fig. 1F–J). To further confirm that the MSC processing procedures induced more heterogenicities than the tissue origin, the primary isolated or in vitro expanded MSCs were labeled. Both PCA and tSNE analysis showed that the in vitro expanded MSCs clustered together much more tightly than the primary isolated MSCs (Fig. 1K, L). Thus, the RNA sequencing data analysis here proposed that the MSC processing procedures might induce more heterogenicities than the tissue origin in the mouse MSCs, indicating that the MSC processing procedures should be standardized in the future. However, the data of the in vitro expanded MSCs were all from the same laboratory (Fig. 1E, K) and contained two different tissue origins (Fig. 1D, K). And this was further confirmed with the tSNE analysis (Fig. 1I, J, L). Therefore, more data and further analysis are needed to confirm this finding.

**Fig. 2:**
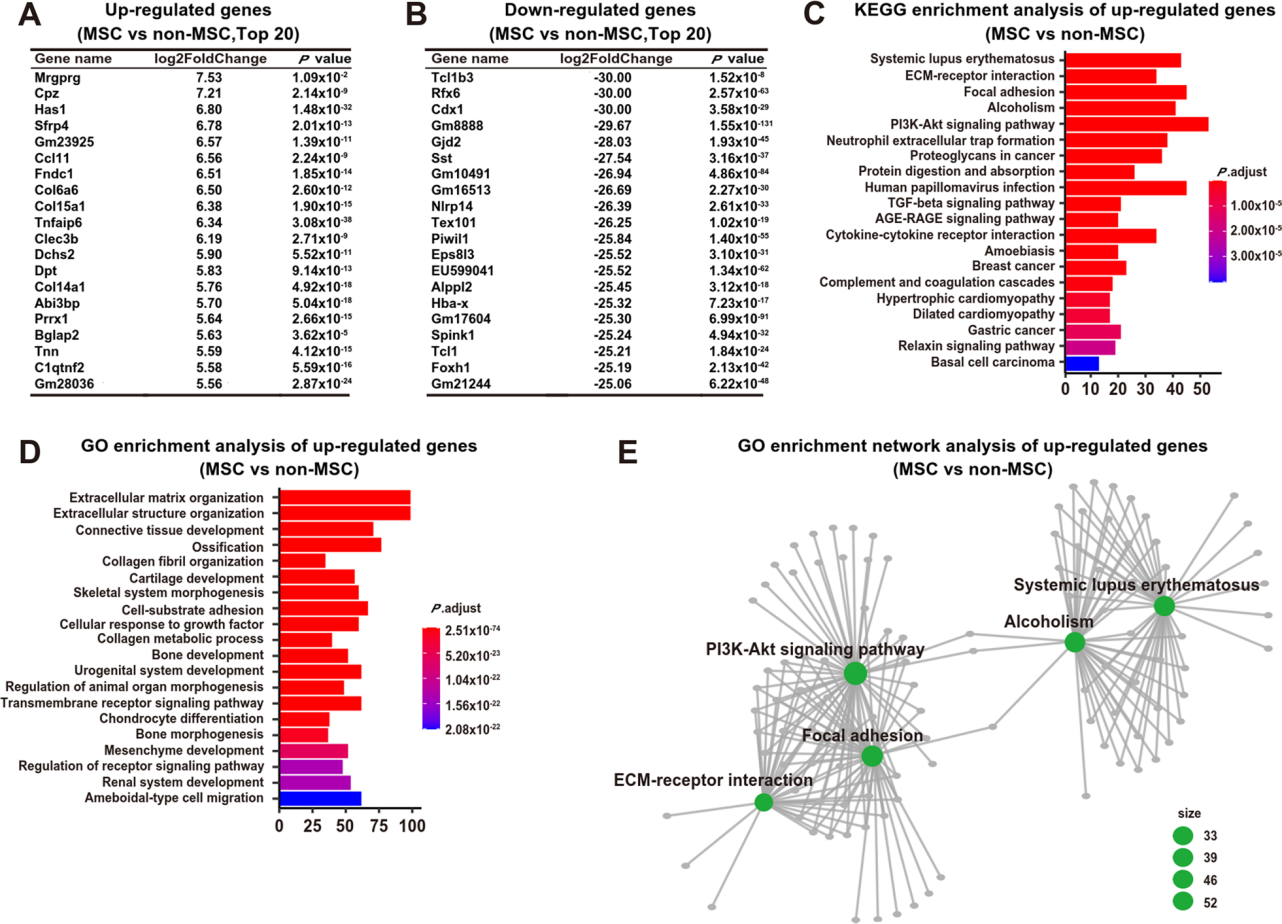
Differential gene expression analysis between MSCs and non-MSCs. **A** Top 20 up-regulated genes in MSCs when comparing with non-MSCs. **B** Top 20 down-regulated genes in MSCs when comparing with non-MSCs. **C** KEGG enrichment analysis of up-regulated genes in MSCs when comparing with non-MSCs. **D** GO enrichment analysis of up-regulated genes in MSCs when comparing with non-MSCs. **E** GO enrichment network analysis of up-regulated genes in MSCs when comparing with non-MSCs. *MSCs* mesenchymal stromal/stem cells. *KEGG* Kyoto Encyclopedia of Genes and Genomes; *GO* gene ontology

Then, the differentially expressed genes (DEG) were analyzed. There were totally 812 up-regulated gene and 6821 down-regulated gene in MSCs when comparing with the non-MSCs with the cutoff as log2FC (log2 fold change) > 2 (Additional file 4: Table S4, Additional file 5: Table S5). Among the top 20 highly expressed genes in MSCs, 2 have immune modulation functions (including Mrgprg and Tnfaip6), and 15 are deposited into ECM (extracellular matrix) or secreted (including Has1, Col6a6, Col15a1, Tnfaip6, Fndc1, Clec3b, Dchs2, Sfrp4, Ccl11, Dpt, Col14a1, Abi3bp, Bglap2, Tnn, and C1qtnf2) (Fig. 2A). Among the top 20 down-regulated genes in MSCs, they have quite diverse functions, including AKT pathway regulators (Tcl1b3 and Tcl1), TGF pathway regulator (Foxh1), EGF pathway regulator (Eps8l3), RNA processing (Piwil1), hormone (Sst), cell communication through tight junction (Gjd2), immune regulation (Nlrp14), metabolic regulation (Alppl2 and Spink1), and differentiation regulations (Rfx6 for islet cell differentiation, Cdx1 for enterocyte differentiation, and Tex101 for testis differentiation) (Fig. 2B). Indeed, KEGG and GO enrichment of up-regulated genes in MSCs revealed that these genes enriched in ECM modulation and immune regulation (Fig. 2C–E). Therefore, the MSCs tend to have ECM modulation and immune regulation functions.

**Fig. 3:**
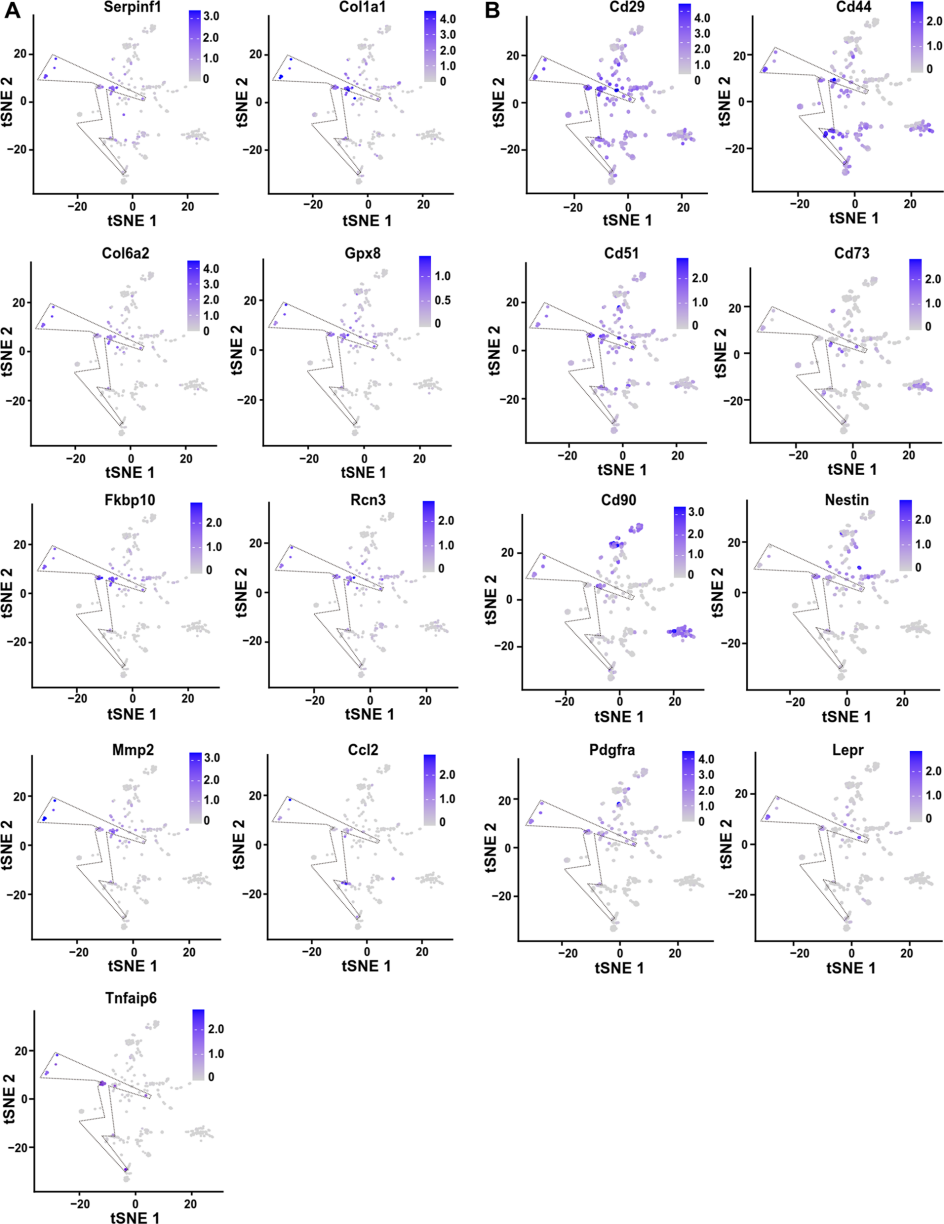
Tnfaip6 is a potential MSC marker—bioinformatic feature plotting. **A** Plotting of potential MSC marker genes. **B** Plotting of known MSC marker genes. *Serpinf1* serpin family F member 1; *Col1a1* collagen type I alpha 1 chain; *Col6a2* collagen type 6 alpha 2 chain; *Gpx8* glutathione peroxidase 8; *Fkbp10* FK506 binding protein prolyl isomerase 10; *Rcn3* reticulocalbin 3; *Mmp2* matrix metallopeptidase 2; *Ccl2* C–C motif chemokine ligand 2; *Tnfaip6* tumor necrosis factor alpha-induced protein 6; *Pdgfra* platelet-derived growth factor receptor alpha; and *Lepr* leptin receptor

Because of the high heterogenicity of MSCs, some potential MSC markers might be neglected by bioinformatic analysis. All these up-regulated genes (Additional file 4: Table S4) in MSCs were plotted by FeaturePlot function in R, based on their expression levels. Then, the plotting was manually screened by 3 investigators independently based on the criteria: highly expressed in MSCs and expressed in MSCs from all origins (including different tissues, different laboratories, and fresh isolated or expanded). Finally, 9 potential markers were identified, including Serpinf1, Col1a1, Fkbp10, Rcn3, Col6a2, Gpx8, Mmp2, Ccl2, and Tnfaip6 (Figs. 1F, 3A, Additional file 6: Table S6). Interestingly, the Tnfaip6 was identified with both bioinformatic analysis (Fig. 2A) and manually screening (Fig. 3A) and exclusively highly expressed in MSCs in the quiescent state (Figs. 1F, 3A). In addition, the known mouse MSC markers were also plotted, including Cd44, Pdgfra, Lepr, Nestin, Cd29, and Cd51 (Fig. 3B). All these known mouse MSC markers were either expressed in non-MSCs, low expressed in MSCs or not expressed in most types of MSCs from all origins (Fig. 3B), indicating that these markers are not MSC specific. Furthermore, the lineage specific genes were not expressed in MSCs, except that the CD34 was expressed in some MSC samples with low levels (Additional file 7: Fig. S1). Therefore, the Tnfaip6 is a potential MSC marker.

**Fig. 4:**
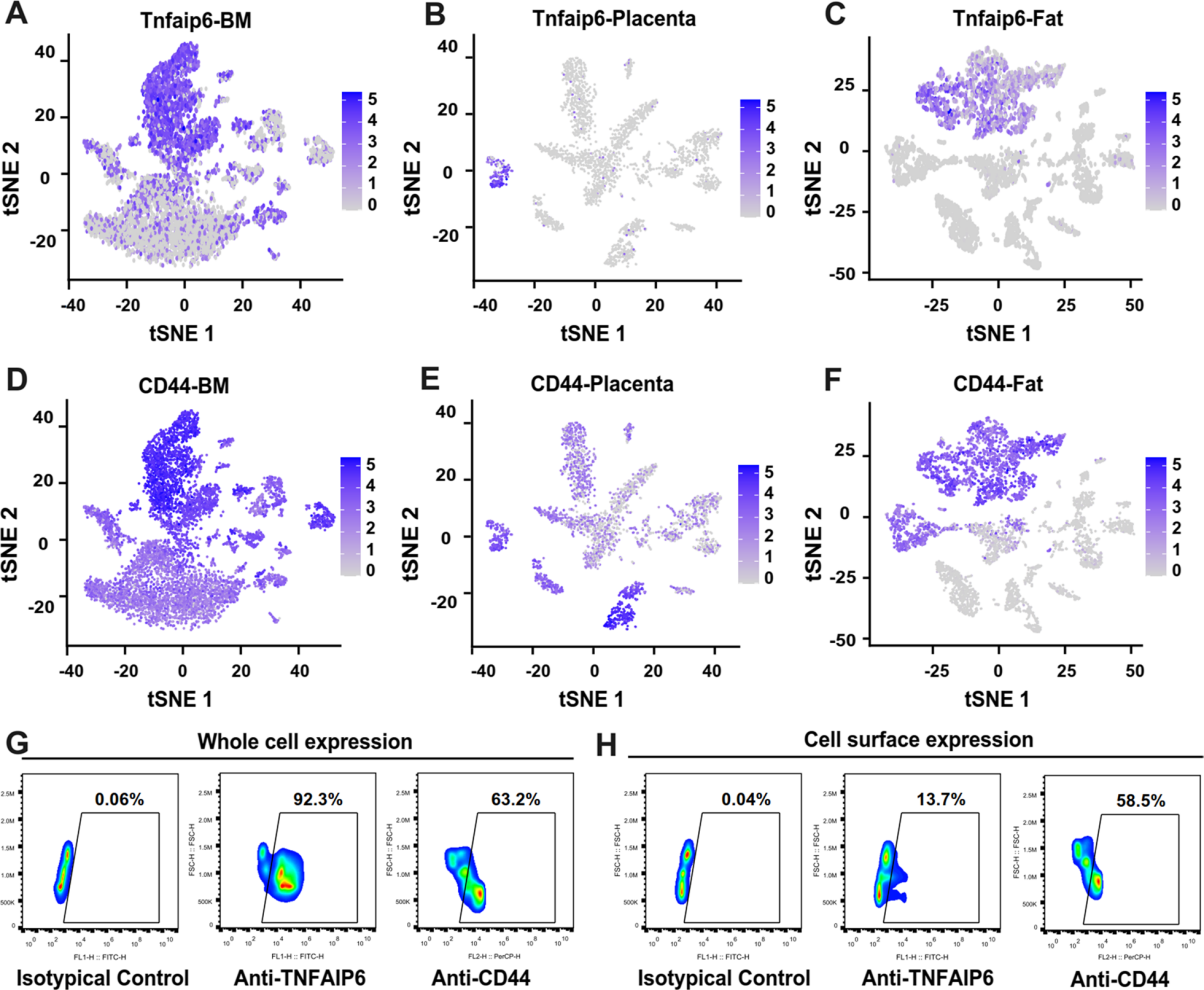
TNFAIP6 is a potential MSC marker-expression validation. **A** The mRNA level of TNFAIP6 was plotted on single-cell RNA sequencing data of bone marrow cells. **B** The mRNA level of TNFAIP6 was plotted on single-cell RNA sequencing data of placenta cells. **C** The mRNA level of TNFAIP6 was plotted on single-cell RNA sequencing data of fat tissue cells. **D** The mRNA level of CD44 was plotted on single-cell RNA sequencing data of bone marrow cells. **E** The mRNA level of CD44 was plotted on single-cell RNA sequencing data of placenta cells. **F** The mRNA level of CD44 was plotted on single-cell RNA sequencing data of fat tissue cells. **G** The protein levels of TNFAIP6 and CD44 were determined by flow cytometry after cell fixation and permeabilization. **H** The cell surface expression levels of TNFAIP6 and CD44 were determined by flow cytometry. *TNFAIP6* tumor necrosis factor alpha-induced protein 6; *BM* bone marrow

To further confirm that the Tnfaip6 is a potential MSC marker uncovered by RNA sequencing data analysis, the fresh isolated cells from bone marrow, placenta, and fat tissues were subjected to single-cell RNA sequencing. Data showed that the Tnfaip6 was expressed in all these three types of tissues (Fig. 4A–C). In the meantime, the expression of mouse MSC marker CD44 was also detected (Fig. 4D–F) [6]. Furthermore, the expression of TNFAIP6 and CD44 was also detected at the protein levels by flow cytometry in the bone marrow cells after fixation and permeabilization (Fig. 4G). However, the cell surface level of TNFAIP6 was dramatically reduced (Fig. 4H), which is in accordance with previous findings that the TNFAIP6 is a secreted protein [64].

**Fig. 5:**
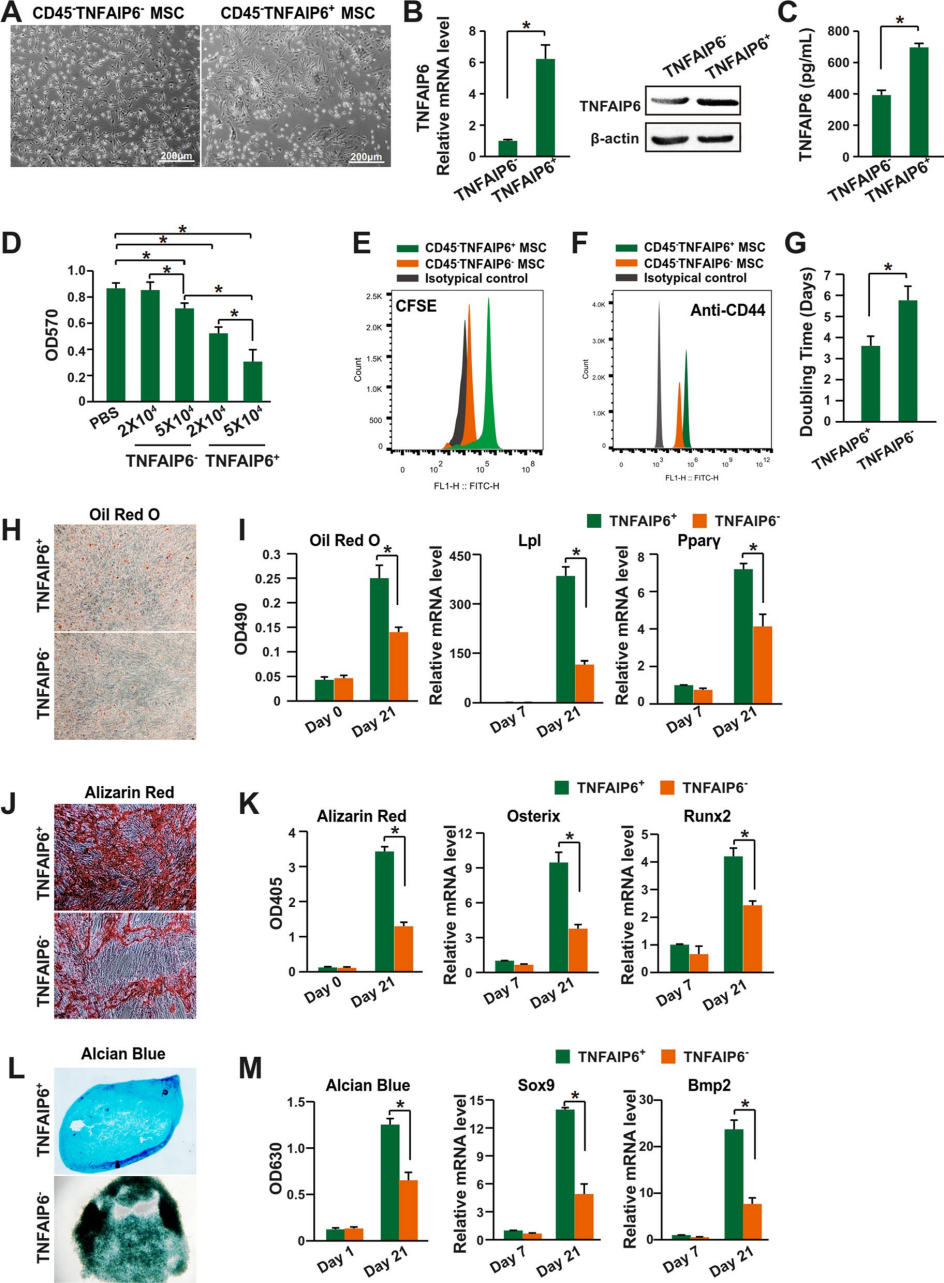
TNFAIP6 defines a subpopulation of mouse MSCs with enhanced immune suppression activities. **A** Cell morphology of CD45^−^TNFAIP6^−^ and CD45^−^TNFAIP6^+^ MSCs. **B** Left panel: mRNA level of TNFAIP6 was assessed by qPCR (n = 3); Right panel: protein level of TNFAIP6 was assessed by western blot. **C** Protein level of TNFAIP6 secreted from the CD45^−^TNFAIP6^−^ or CD45^−^TNFAIP6^+^ MSCs (n = 3). **D** Splenocytes proliferation assay after co-culture with CD45^−^TNFAIP6^−^ or CD45^−^TNFAIP6^+^ MSCs (n = 3). Both high cell number (5 × 10^4^) and low cell number (2 × 10^4^) were assessed. **E** Splenocytes proliferation assay after co-culture with CD45^−^TNFAIP6^−^ or CD45^−^TNFAIP6^+^ MSCs was assessed with CFSE approach. High cell number (5 × 10^4^) was used. **F** CD44 expression of CD45^−^TNFAIP6^−^ or CD45^−^TNFAIP6^+^ MSCs, determined by flow cytometry. **G** Cell proliferation of CD45^−^TNFAIP6^−^ or CD45^−^TNFAIP6^+^ MSCs was determined by doubling time (n = 3). **H** The adipocytes differentiation efficiency was assessed by Oil Red O staining. **I** The adipocytes differentiation efficiency was quantified by Oil Red O staining and qPCR analysis of gene Lpl and Pparγ (n = 3). **J** The osteocytes differentiation efficiency was assessed by Alizarin Red staining. **K** The osteocytes differentiation efficiency was quantified by Alizarin Red staining and qPCR analysis of gene Osterix and Runx2 (n = 3). **L** The chondrocytes differentiation efficiency was assessed by Alcian Blue staining. **M** The chondrocytes differentiation efficiency was quantified by Alcian Blue staining and qPCR analysis of gene Sox9 and Bmp2 (n = 3). * indicates *P* < 0.05. *TNFAIP6* tumor necrosis factor alpha-induced protein 6; *MSCs* mesenchymal stromal/stem cells; *Lpl* lipoprotein lipase; *Pparγ* peroxisome proliferator-activated receptor gamma; *Osterix* Sp7 transcription factor; *Runx2* RUNX family transcription factor 2; *Sox9* SRY-box transcription factor 9; and *Bmp2* bone morphogenetic protein 2

Then, the TNFAIP6^+^ MSCs were purified with FACS (fluorescence-activated cell sorting) after eliminating the CD45^+^ lymphocytes with MACS (magnetic-activated cell sorting). The CD45^−^TNFAIP6^+^ and CD45^−^TNFAIP6^−^ MSCs showed different morphology (Fig. 5A). And the CD45^−^TNFAIP6^+^ MSCs expressed higher levels of TNFAIP6 at both mRNA and protein levels (Fig. 5B, C). They suppressed the lymphocytes proliferation more significantly with dose-dependent effects (Fig. 5D, E). It has been demonstrated that the TNFAIP6 is one of the key factors to maintain the stem cell characteristics of mouse MSCs [76], and thus, we further compared the stem cell characteristics between the CD45^−^TNFAIP6^+^ and CD45^−^TNFAIP6^−^ MSCs. The data showed that the CD45^−^TNFAIP6^+^ MSCs had higher level of CD44 expression (Fig. 5F), proliferation rate (Fig. 5G), and the efficiency of differentiating into adipocytes (Fig. 5H, I), osteocytes (Fig. 5J, K), and chondrocytes (Fig. 5L, M). Therefore, the TNFAIP6^+^ MSCs had enhanced immune suppression activities.

**Fig. 6:**
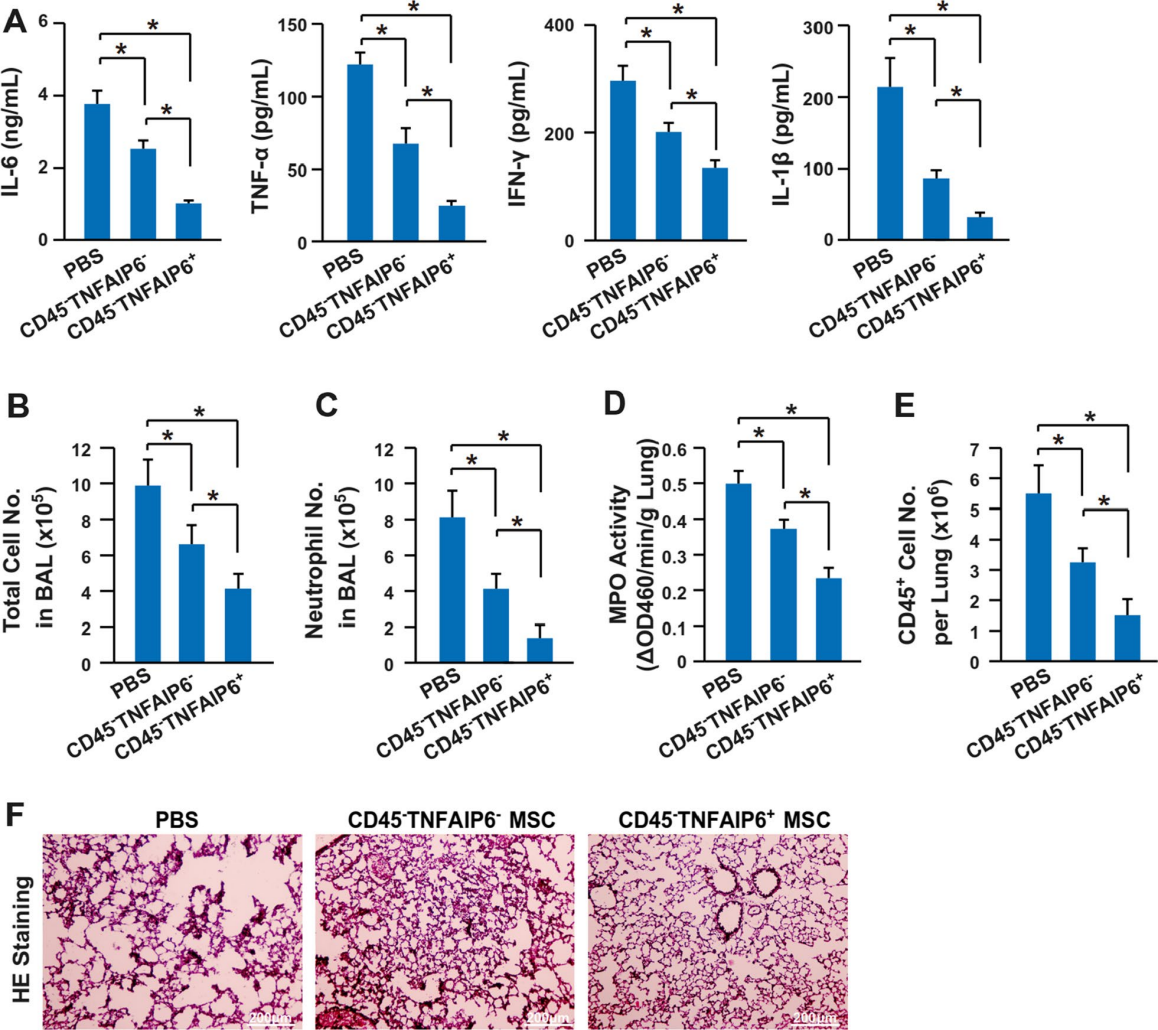
TNFAIP6^+^ MSCs have enhanced anti-inflammatory activities in the mouse model of acute inflammation. **A** Serum level of IL-6, TNF-α, IFN-γ, and IL-1β was determined after 24 h post-LPS stimulation via ELISA (n = 8). **B** The total cell number in BAL was determined after 24 h post-LPS stimulation by flow cytometry (n = 8). **C** The neutrophil number in BAL was determined as CD45^+^CD11b^+^Ly-6G^+^Ly-6C^med^ after 24 h post-LPS stimulation by flow cytometry (n = 8). **D** The MPO activity was quantified after 24 h post-LPS stimulation (n = 8). **E** The CD45^+^ cells in the lung were measured after 7 days post-LPS stimulation flow cytometry (n = 8). **F** Representative figures for HE staining of lung tissues after 7 days post-LPS stimulation. * indicates *P* < 0.05. *TNFAIP6* tumor necrosis factor alpha-induced protein 6; *IL-6* interleukin 6; *TNF-α* tumor necrosis factor alpha; *IFN-γ* interferon gamma; *IL-1β* interleukin 1 beta; *BAL* bronchoalveolar lavage; and *MPO* myeloperoxidase

**Fig. 7:**
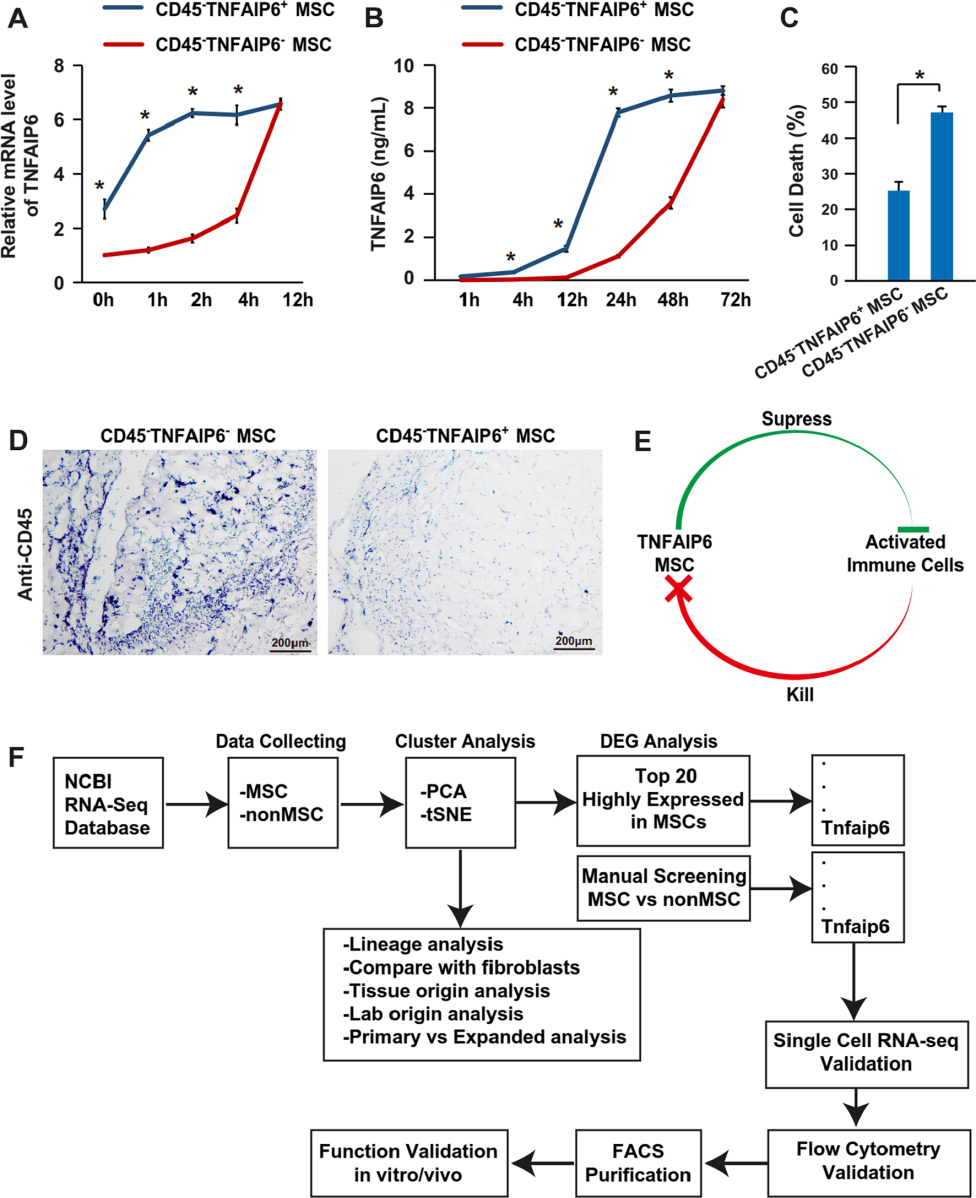
TNFAIP6^+^ MSCs are resistant to immunologic cytotoxicity through faster response to immune stimulation. **A** The mRNA level of TNFAIP6 was determined via qPCR after stimulated with 10 ng/mL TNF-α, 10 ng/mL IL-1β, and 10 ng/mL IFN-γ for 48 h (n = 3). **B** The protein level of TNFAIP6 was determined via ELISA after stimulated with 10 ng/mL TNF-α, 10 ng/mL IL-1β, and 10 ng/mL IFN-γ for 48 h (n = 3). **C** Cytotoxicity was detected by measuring LDH secretion after co-culture with stimulated splenocytes (n = 3). **D** Representative lymphocytes infiltration within in the Matrigel plug containing TNFAIP6^−^ or TNFAIP6^+^ MSCs at day 3 post-cell transplantation. **E** Proposed potential mechanism of enhanced immune suppression activities of TNFAIP6^+^ MSCs. **F** Representation of the process to identify the TNAFAIP6 as a MSC marker. * indicates *P* < 0.05. *TNFAIP6* tumor necrosis factor alpha-induced protein 6; *MSCs* mesenchymal stromal/stem cells; and *LDH* lactate dehydrogenase

To further validate the enhanced immune suppression function of TNFAIP6^+^ MSCs in vivo, the mouse model of acute inflammation was established via intraperitoneal injection of LPS. Indeed, the TNFAIP6^+^ MSCs reduced the serum levels of pro-inflammatory cytokines (IL-6, TNF-α, IFN-γ, and IL-1β) (Fig. 6A), total cell number (Fig. 6B), and neutrophil infiltration (Fig. 6C, D) more significantly than the TNFAIP6^−^ MSCs at 24 h post-LPS stimulation. Furthermore, the TNFAIP6^+^ MSCs promoted the lung recovery through reducing lymphocytes infiltration (Fig. 6E) and maintaining tissue structure (Fig. 6F) more significantly than the TNFAIP6^−^ MSCs at day 7 after LPS stimulation. The TNFAIP6 could be induced by pro-inflammatory factors, such as TNF-α, IL-1β, IFN-γ, and LPS [57, 63–65]. Therefore, both TNFAIP6^+^ and TNFAIP6^−^ MSCs should express TNFAIP6 in *vivo* after LPS stimulation. To uncover the underlying mechanism that the TNFAIP6^+^ MSCs had enhanced immune suppression activities, the TNFAIP6^+^ and TNFAIP6^−^ MSCs were stimulated with pro-inflammatory cytokines (10 ng/mL TNF-α, 10 ng/mL IL-1β, and 10 ng/mL IFN-γ) to mimic the inflammatory environment. Interestingly, the TNFAIP6^+^ MSCs responded the stimulation more quickly than the TNFAIP6^−^ MSCs at both mRNA and protein levels (Fig. 7A, B). Previously, we demonstrated that the anti-inflammatory cytokine IL-37 could improve the therapeutic effects of MSCs through promoting their survival in vivo [5, 6]. Therefore, we hypothesized that the TNFAIP6^+^ MSCs might also have better survival in vivo through its anti-inflammatory activities. Indeed, co-culture assay showed that the TNFAIP6^+^ MSCs had higher level of cell survival and lower cell death induced by stimulated splenocytes (Fig. 7C). Then, the TNFAIP6^+^ or TNFAIP6^−^ MSCs were mixed with Matrigel and injected intraperitoneally. The data showed that the TNFAIP6^+^ MSCs recruited less lymphocytes than TNFAIP6^−^ MSCs (Fig. 7D). Therefore, the TNFAIP6^+^ MSCs might respond to the inflammatory environment and secrete the anti-inflammatory cytokine TNFAIP6 more quickly, resulting in immediately immune suppression and less MSC death induced by activated immune cells. The survived MSCs express more anti-inflammatory cytokines and further enhance the immune suppression function (Fig. 7E). In summary, we demonstrated that the TNFAIP6^+^ MSCs had enhanced immune suppression capabilities (Fig. 7F).

## Discussion

The therapeutic inconsistency of MSCs significantly hampers their large-scale clinical applications [1–3, 8]. Therefore, reducing the MSC heterogenicity and improving their therapeutic consistency are critical and urgently needed [1, 4, 8]. Among different strategies, identifying and purifying specific MSC subpopulation with specific functions is a promising strategy to reduce the MSC heterogenicity and improve their therapeutic consistency [1]. Through lineage tracing and function validation, many different makers were identified for the mouse MSCs [13–47]. However, these markers are not MSC specific. And most of them are also not related to their functions and therapeutic efficacy, or the underlying mechanism linking the MSC marker to the function is missing. Therefore, identifying and purifying specific MSC subpopulation with specific function is necessary for their therapeutic applications.

In the current study, the purified CD45^−^TNFAIP6^+^ mouse MSCs showed enhanced immune suppression activities and improved therapeutic effects in the mouse model of acute inflammation. Although direct application of recombinant TNFAIP6 protein also has significant therapeutic effects in several mouse models [75, 77–80], similar to our previous investigations on the therapeutic effects of IL-37[6], the TNFAIP6 protein also has a very short half-life in vivo (around 0.2 h) [77]. The TNFAIP6^+^ MSCs respond to the inflammatory environment and secrete the anti-inflammatory cytokine TNFAIP6 more quickly, resulting in immediately immune suppression and less MSC death induced by activated immune cells. And the survived MSCs express high level of anti-inflammatory cytokines, which further enhances the immune suppression function as positive feedback.

## Conclusions

In conclusion, we have identified TNFAIP6 as a new MSC marker and the TNFAIP6^+^ MSC subpopulation has enhanced immune suppression capabilities.

## Supplementary Information

**Additional file 1**: Basic information of non-MSC samples.

**Additional file 2**: Basic information of MSC samples.

**Additional file 3**: qPCR primer sequences.

**Additional file 4**: Up-regulated genes in MSC vs non-MSC (FC > 2).

**Additional file 5**: Down-regulated genes in MSC vs non-MSC (FC > -2).

**Additional file 6**: Manual screening daa.

**Additional file 7: Figure 1**. Plotting of MSC negative marker genes.

## Data Availability

The datasets for this study are available on request from the corresponding author.

## Abbreviations

MSCs: Mesenchymal stromal/stem cells
TNFAIP6: Tumor necrosis factor alpha-induced protein 6
PCA: Principal component analysis
tSNE: t-distributed stochastic neighbor embedding
GO: Gene ontology
KEGG: Kyoto Encyclopedia of Genes and Genomes
BM: Bone marrow
FBS: Fetal bovine serum
bFGF: Basic fibroblast growth factor
PFA: Paraformaldehyde
BAL: Bronchoalveolar lavage
MPO: Myeloperoxidase
HE: Hematoxylin and eosin
DEG: Differentially expressed genes
Log2FC: Log2 fold change
ECM: Extracellular matrix
