# Supplementary figures and images for: TNFAIP6 defines the MSC subpopulation with enhanced immune suppression activities
Source: Stem Cell Res Ther. 2022 Sep 24;13:479. doi: 10.1186/s13287-022-03176-5 (PMC9509641; doi:10.1186/s13287-022-03176-5)

Supplementary Figure 1

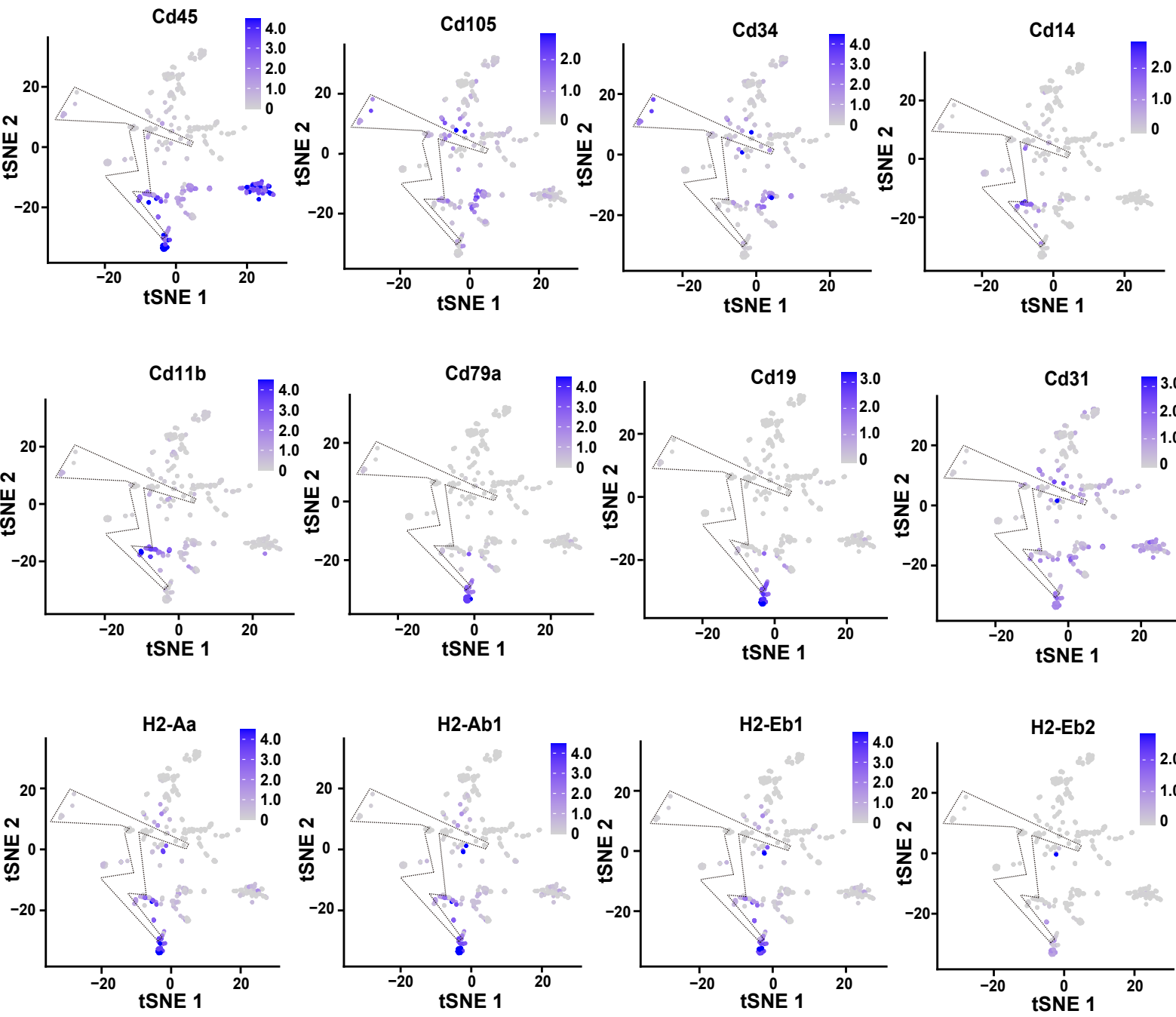

Supplement: Supplementary file 7 — Additional file 7: Figure 1. Plotting of MSC negative marker genes. [file 13287_2022_3176_MOESM7_ESM.pdf]
